# Alternative Polyadenylation Drives Runaway Pro‐Inflammatory Macrophages in Periodontitis by Enabling Escape From miRNA Repression

**DOI:** 10.1111/cpr.70156

**Published:** 2025-12-22

**Authors:** Jing Zhang, Yilong Zhao, Jiaru Deng, Shuyuan Qu, Yiyi Zhou, Qin Zhao, Yufeng Zhang

**Affiliations:** ^1^ State Key Laboratory of Oral & Maxillofacial Reconstruction and Regeneration, Key Laboratory of Oral Biomedicine Ministry of Education, Hubei Key Laboratory of Stomatology, School & Hospital of Stomatology Wuhan University Wuhan China; ^2^ Medical Research Institute School of Medicine, Wuhan University Wuhan China; ^3^ Taikang Center for Life and Medical Sciences Wuhan University Wuhan China

**Keywords:** alternative polyadenylation, macrophage polarisation, miRNA repression, periodontitis, post‐transcriptional regulation

## Abstract

Periodontitis is a chronic inflammatory disease driven by a dysregulated host immune response, in which macrophage‐mediated inflammation shifts from protective to pathological. While monocyte‐derived macrophages (MDMs) are known to adopt a destructive, M1‐like pro‐inflammatory phenotype, the mechanisms that enable this ‘runaway’ polarisation by bypassing endogenous negative feedback remain elusive. Here, we identify alternative polyadenylation (APA) as a critical post‐transcriptional mechanism driven by pathogens to disrupt macrophage immune control. Integrating single cell RNA sequencing with Sierra APA analysis of human gingival tissues, we uncovered a global shift toward proximal poly(A) site (PAS) usage, indicative of 3′UTR shortening, specifically within the pro‐inflammatory MDM subset. This APA remodelling preferentially affected genes essential for cytokine production and inflammatory signalling. In vitro, the keystone pathogen *Porphyromonas gingivalis* similarly induced widespread 3′UTR shortening in macrophages. This shortening systematically eliminated inhibitory miRNA‐binding sites, thereby derepressing pro‐inflammatory transcripts. Mechanistically, using *Selenok* as a representative example, we demonstrate that *P. gingivalis* induced 3′UTR shortening selectively abolishes repression by miR‐320‐3p, a ‘brake’ miRNA upregulated in periodontitis, whose binding site is excised by the proximal APA event. Collectively, these findings reveal APA remodelling as a key pathogenic strategy that enables pro‐inflammatory macrophages to escape miRNA‐mediated suppression, leading to an uncontrolled M1‐like state. This ‘disruption’ of the post‐transcriptional braking system provides a new mechanistic rationale for the persistent, destructive inflammation in periodontitis.

## Graphical abstract

APA‐Guided Therapeutic miRNA Selection for Periodontitis.

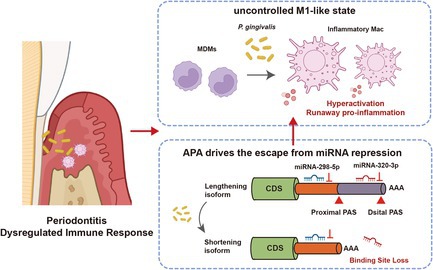

## 1 Introduction

Periodontitis is a chronic inflammatory disease initiated by pathogenic infection but sustained by a profoundly dysregulated host immune response [1, 2]. This immune dysregulation manifests as a pathological shift from protective host defence to destructive, self‐perpetuating inflammation [3, 4, 5]. Macrophages, central orchestrators of the innate immune response, are pivotal drivers of this destructive shift [6, 7, 8]. Within the diseased periodontal microenvironment, infiltrating monocyte‐derived macrophages (MDMs) adopt a hyper‐inflammatory, M1‐like phenotype, becoming key effectors of tissue destruction through the persistent release of pro‐inflammatory cytokines and matrix‐degrading enzymes [9, 10].

A healthy immune response is critically dependent on negative feedback loops, or ‘braking mechanisms’, that ensure inflammation is resolved promptly after pathogen clearance to prevent collateral tissue damage [11, 12, 13]. Post‐transcriptionally, microRNAs (miRNAs) are essential components of this braking system, serving to fine‐tune and terminate inflammatory gene expression [14, 15, 16]. This presents a core pathological paradox in periodontitis: if these miRNA‐mediated brakes exist, why does the M1 macrophage pro‐inflammatory program become uncontrolled, persistent, and ‘runaway’? This strongly implies that a mechanism must be active within these macrophages that allows key inflammatory genes to systematically circumvent or escape this endogenous miRNA repression.

Alternative polyadenylation (APA) is a potent post‐transcriptional mechanism uniquely capable of globally rewiring the miRNA regulatory landscape [17, 18]. By selecting alternative polyadenylation sites (PAS), APA generates mRNA isoforms with distinct 3′ untranslated region (3′UTR) lengths [19, 20, 21]. Specifically, the selection of a proximal PAS results in 3′UTR shortening, a mechanism that physically excises miRNA‐binding sites predominantly located in the distal 3′UTR [22, 23]. This strategy is exploited by proliferating cells, such as activated T cells and cancer cells, to ‘escape’ miRNA suppression and sustain high protein output [24, 25]. However, whether this APA‐mediated regulatory ‘disruption’ is driven by pathogens to disable macrophage immune brakes and drive runaway M1 polarisation in chronic infection remains unknown.

Here, we hypothesize that the keystone periodontal pathogen *Porphyromonas gingivalis* ( *P. gingivalis* ) [26, 27, 28] actively induces systemic APA remodelling in macrophages. We posit that this remodelling causes widespread 3′UTR shortening of key pro‐inflammatory genes, thereby facilitating their escape from host miRNA‐mediated suppression. This post‐transcriptional ‘derepression’, we propose, is a key mechanism driving the uncontrolled M1 macrophage polarisation that underpins the chronic destructive inflammation of periodontitis. To test this, we integrated single‐cell RNA sequencing (scRNA‐seq) of human gingival tissues with the Sierra APA analysis framework to map in vivo APA dynamics at single‐cell resolution [29]. We then used *P. gingivalis* ‐infected bone marrow–derived macrophages (BMDMs) to confirm the pathogen's instructive role in vitro. Our study not only identifies a pro‐inflammatory macrophage subset (inflammatory mac) defined by global APA shortening but also provides a precise molecular dissection, using *Selenok* as a representative example, of how APA severs the specific ‘brake line’ of miR‐320‐3p to unleash gene expression. This work uncovers a novel layer of macrophage dysregulation, invisible to conventional expression or RNA velocity analyses, and offers new mechanistic insights into the pathogenesis of chronic inflammatory disease.

## 2 Results

### 2.1 Single‐Cell Landscape Reveals Macrophage Expansion as a Hallmark of Periodontitis

**FIGURE 1:**
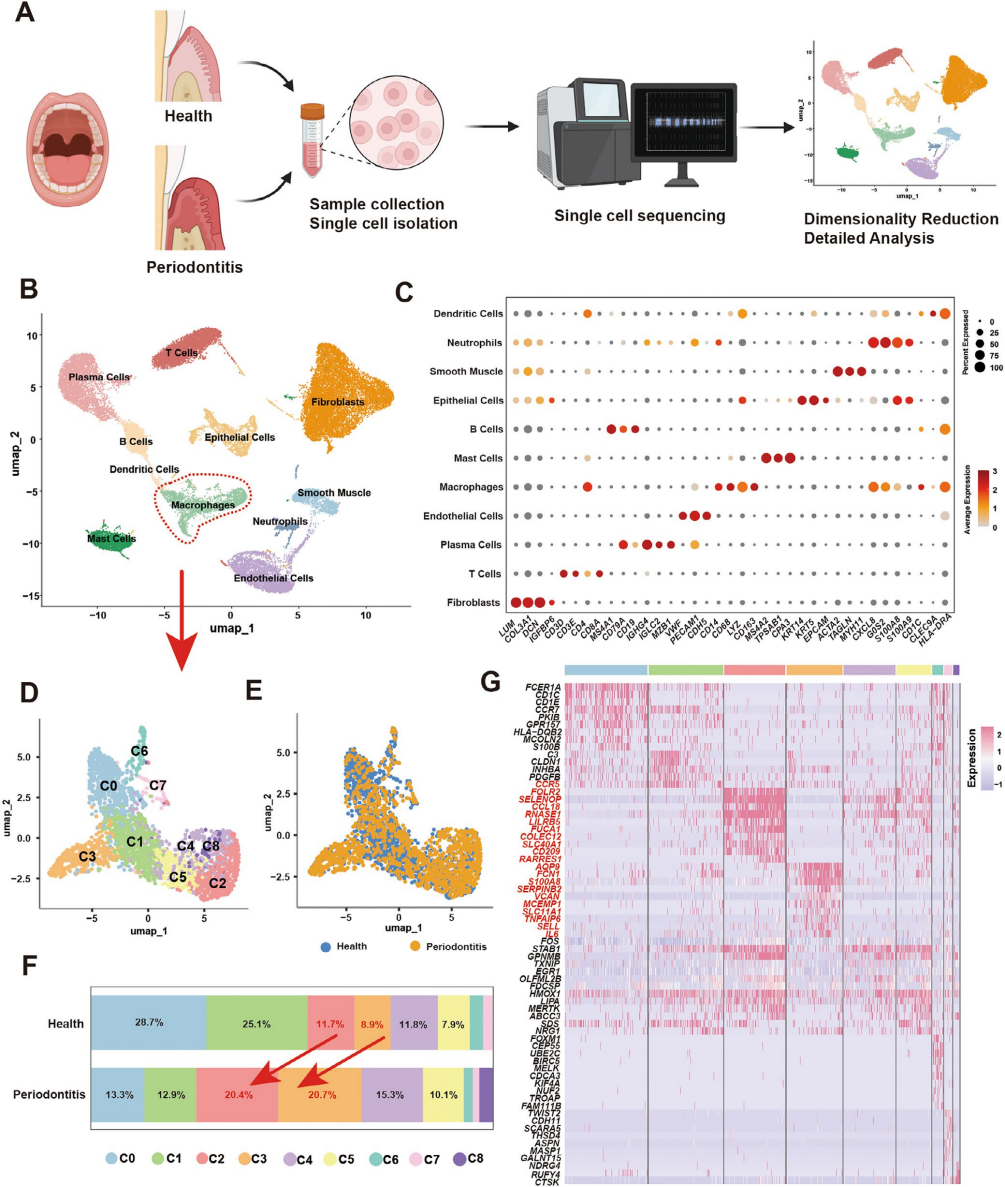
Single‐cell landscape reveals macrophage expansion as a hallmark of periodontitis. (A) Schematic diagram illustrating single‐cell RNA sequencing analysis of gingival tissue samples from periodontitis patients and healthy volunteers. (B) UMAP plot of all cell clusters, with macrophages circled in red dashed lines. (C) Dot plot displaying the expression of characteristic genes from different cell clusters. (D) UMAP plot presenting further cell clustering of macrophages from (B). (E) UMAP plot of macrophages from the healthy and periodontitis groups. (F) Cell proportion of macrophage subpopulations in the healthy and periodontitis groups. Red arrows indicate that the cell proportion in clusters 2 and 3 is significantly elevated in the periodontitis group. (G) Heatmap displaying the top 10 differentially expressed marker genes across macrophage subpopulations.

To comprehensively delineate the immune landscape of gingival tissues under chronic bacterial challenge, we performed scRNA‐seq on gingival biopsies from periodontitis patients and healthy controls (Figures 1A and S1A, *n* = 3). After stringent quality control and correction for batch‐related variation, UMAP projection resolved major immune and stromal compartments, reflecting the intricate cellular composition of the gingival microenvironment (Figure 1B). Cell‐type annotation was confirmed using canonical lineage markers (Figures 1C and S1B,E). Quantitative cell composition analysis revealed a striking expansion of macrophages in periodontitis samples compared to healthy gingiva (Figure S1C,D), suggesting enhanced recruitment or local proliferation in response to persistent microbial challenge. Consistent with this, cell–cell communication analysis showed that macrophages exhibited the highest interaction strength among immune populations (Figure S1F,G), with particularly strong IL6‐mediated signalling (Figure S1H). These findings establish macrophages as central hubs of immune crosstalk and inflammatory amplification in the diseased gingiva, providing the rationale for focusing on their functional reprogramming in subsequent analyses.

Macrophages displayed pronounced transcriptional heterogeneity, indicative of functional specialisation in response to microbial stress [30]. Reclustering of macrophage populations resolved nine transcriptionally distinct subclusters, each characterised by unique marker signatures (Figure 1D,E). Among them, C2 and C3 clusters were markedly expanded in periodontitis tissues (Figure 1F), suggesting monocyte‐derived populations that are locally recruited or proliferate under chronic infection, acquiring specialised effector functions within the inflammatory milieu. The heatmap further illustrated distinct yet partially overlapping transcriptional programs among these subsets, reflecting functional diversification built upon shared developmental origins (Figure 1G).

### 2.2 Identification of a Pro‐Inflammatory M1‐Like Macrophage Subset (Inflammatory Mac) Driving Inflammation

**FIGURE 2:**
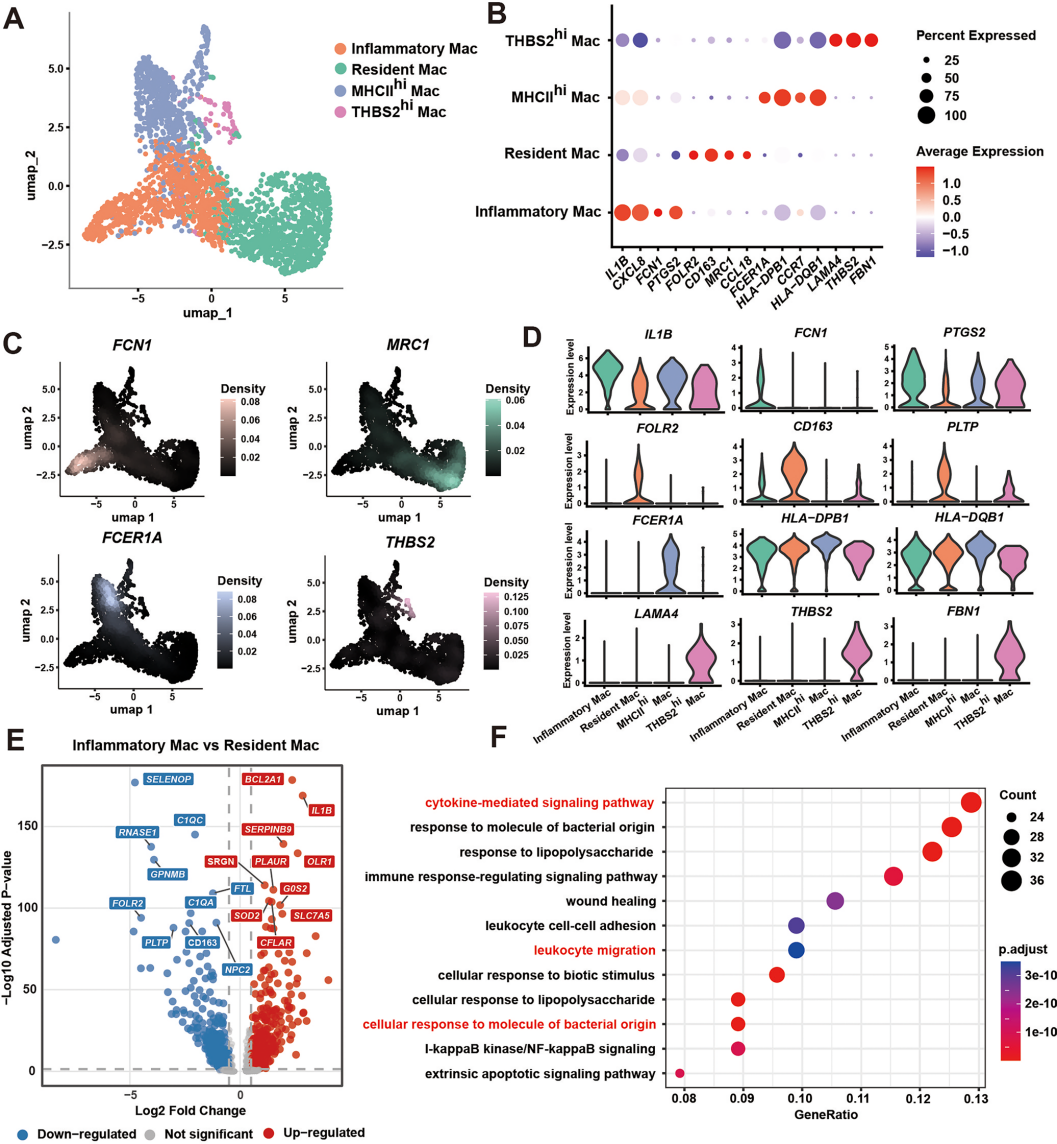
Identification of a pro‐inflammatory M1‐like macrophage subset (inflammatory mac) driving inflammation. (A) UMAP plot of macrophage subset annotation. (B) Dot plot displaying the expression of characteristic genes from different macrophage subsets. (C) UMAP density plot of representative marker gene expression across macrophage subsets. (D) Violin plot of marker gene expression across macrophage subsets. (E) Volcano plot of differentially expressed genes between inflammatory mac and resident mac, with red and blue labels indicating the top 10 up‐regulated and down‐regulated genes respectively. (F) GO enrichment plot for genes upregulated in (E).

To further define macrophage heterogeneity and infer functional specialisation, we re‐annotated macrophage subsets at higher resolution (Figures 2A and S2A). UMAP visualisation and canonical marker profiling delineated two predominant lineages: tissue‐resident macrophages (resident mac) and monocyte‐derived pro‐inflammatory macrophages (inflammatory mac). Feature plots and dot plots (Figure 2B,D) showed that resident mac expressed high levels of *FOLR2, CD163*, and *MRC2*, consistent with an anti‐inflammatory, tissue‐reparative phenotype typical of long‐lived macrophages maintaining homeostasis. In contrast, inflammatory mac were characterised by elevated expression of *IL1B, CXCL8, FCN1*, and *PTGS2*, consistent with a classical M1‐like transcriptional profile associated with cytokine storm–like activation. These cells displayed strong enrichment for GO terms such as ‘cytokine‐mediated signaling pathway’, ‘response to bacterium’, and ‘regulation of inflammatory response’ (Figure 2F), reinforcing their identity as the primary drivers of inflammation in periodontitis.

Besides these two dominant lineages, minor subsets expressing *HLA‐DPB1* (MHCII^hi^ Mac) and *THBS2* (THBS2^hi^ Mac) were also detected (Figure 2C and S2A), possibly reflecting antigen‐presenting and matrix‐remodelling functions. However, their roles were not further explored in this study. Differential gene expression analysis between inflammatory mac and resident mac identified 712 significantly altered genes (adjusted *p* < 0.05, |log_2_FC| > 0.5) (Figure 2E). Downregulated pathways in the inflammatory subset were enriched for ‘antigen presentation’, ‘immune regulation’, and ‘endocytic recycling’, consistent with the loss of homeostatic control.

Together, these results reveal that macrophage heterogeneity in periodontitis arises from distinct differentiation and activation trajectories rather than uniform activation states. Resident macrophages sustain immune balance, whereas monocyte‐derived inflammatory macrophages undergo profound transcriptional remodelling to sustain inflammatory activation. Building upon this framework, we next investigated how post‐transcriptional mechanisms such as APA enable these inflammatory macrophages to bypass endogenous miRNA‐mediated repression, driving runaway polarisation in chronic infection.

### 2.3 RNA Velocity Reveals a ‘Runaway’ Trajectory Toward the Inflammatory Mac State

**FIGURE 3:**
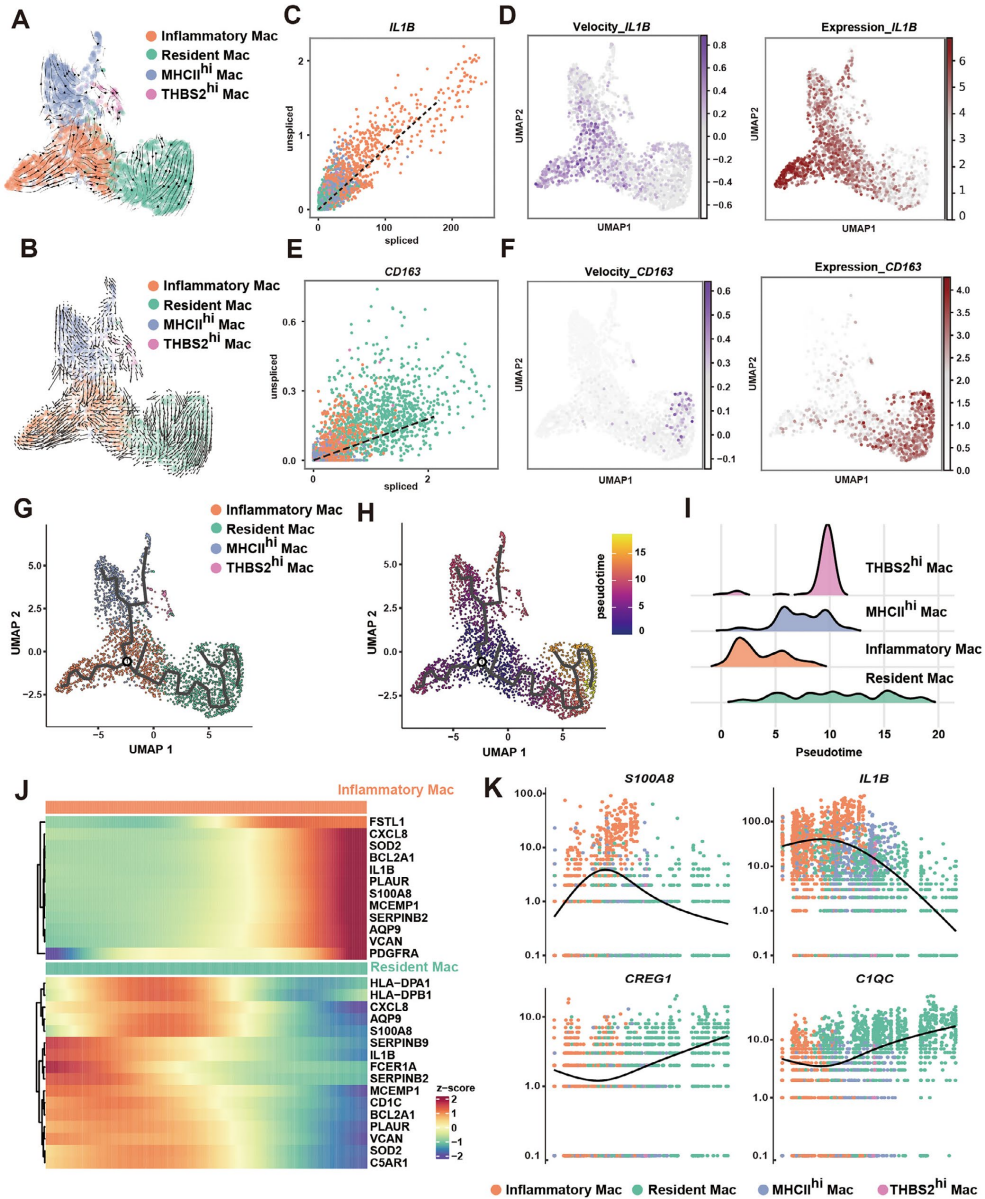
RNA velocity reveals a “runaway” trajectory toward the inflammatory mac state. (A) and (B) The stream plot projected to UMAP illustrating the RNA velocity direction of macrophage subsets. (C) Scatter plot of *IL1B* RNA velocity showing the relationship between unspliced and spliced RNA transcripts across different macrophage subsets. The dashed line indicates the expected steady‐state ratio. (D) UMAP plot of RNA velocity (left) and gene expression (right) of *IL1B*. (E) Scatter plot of *CD163* RNA velocity showing the relationship between unspliced and spliced RNA transcripts across different macrophage subsets. The dashed line indicates the expected steady‐state ratio. (F) UMAP plot of RNA velocity (left) and gene expression (right) of *CD163*. (G) Monocle 3‐based trajectory illustrating the distribution of macrophage subsets. (H) Pseudotime ordering of individual cells, reflecting their progression along the inferred differentiation axis. (I) Evolutionary relationship among macrophage subsets, indicating potential transition paths. (J) Heatmap of top genes dynamically regulated along pseudotime in inflammatory mac and resident mac. (K) Representative gene expression trends along pseudotime, with the *x*‐axis representing pseudotime and the *y*‐axis showing normalised expression levels. Colours denote different subsets.

To delineate the developmental hierarchy and transcriptional dynamics of macrophage subsets, we applied RNA velocity [31] and pseudotime analysis to the macrophage compartment. Velocity streamlines projected onto the UMAP embedding revealed a unidirectional flow originating from monocyte‐like intermediates and converging toward the Inflammatory Mac population (Figure 3A,B). Notably, velocity vectors within cluster 3 of Inflammatory Mac were both longer and denser, indicating accelerated transcriptional turnover and robust RNA processing dynamics. This directional pattern suggested that monocyte‐derived macrophages undergo a ‘runaway’ polarisation trajectory, continuously reinforcing an inflammatory gene program rather than returning to homeostatic equilibrium. Consistent with this kinetic signature, Inflammatory Mac exhibited high expression of hallmark pro‐inflammatory genes including *IL1B* and *S100A8*, as validated by RNA velocity phase portraits showing ongoing transcriptional activation (Figure 3C,D and S3A,B). Resident Mac exhibited high expression of M2 polarisation genes including *CD163* and *FOLR2*, as validated by RNA velocity phase portraits (Figures 3E,F and S3C,D). Pseudotime ordering further revealed a bifurcated lineage structure separating inflammatory mac and resident mac (Figure 3G,I). Along this pseudotime axis, genes involved in innate immune activation and cytokine production were progressively upregulated, whereas homeostatic, antigen‐processing, and matrix‐remodelling programs were gradually repressed (Figure 3J,K). Together, these data illustrate a directional and self‐sustaining differentiation trajectory, in which macrophages dynamically transition into a persistent M1‐like inflammatory state under chronic microbial stress.

However, while RNA velocity captures the momentum of transcriptional change, it remains blind to the isoform‐level regulation that may drive such persistent activation [32, 33]. Standard differential expression analyses measure total gene abundance but overlook alternative mRNA 3′ end processing events that fine‐tune transcript isoform diversity. APA represents a key post‐transcriptional mechanism that remodels 3′UTR length, thereby altering transcript stability, miRNA accessibility, and translation potential, factors crucial for cell fate stabilisation and functional adaptation [34, 35, 36]. These findings prompted us to hypothesize that APA remodelling may act as a molecular lever enabling macrophages to escape inhibitory miRNA‐mediated feedback and sustain pro‐inflammatory activation in periodontitis.

### 2.4 Sierra Analysis Identifies Global 3′UTR Shortening in Pro‐Inflammatory M1‐Like Macrophages

**FIGURE 4:**
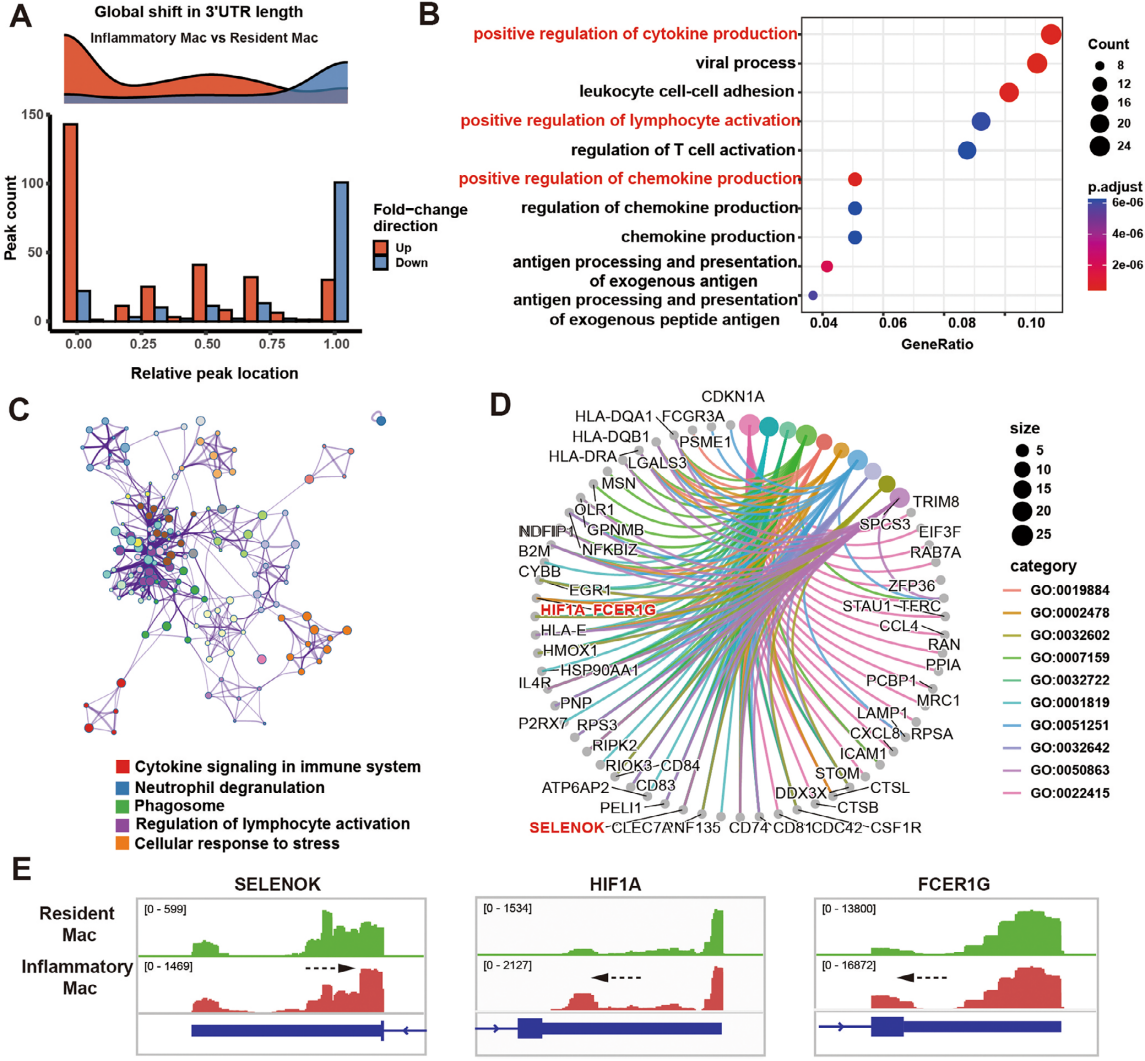
Sierra analysis identifies global 3′UTR shortening in pro‐inflammatory M1‐like macrophages. (A) Counts of 3′UTR peaks showing differential usage according to their relative location to the terminating exon. Location of 0 indicates the peak most proximal to the terminating exon, with 1 representing the most distal. Comparisons performed are for inflammatory mac and resident mac. (B) GO enrichment plot of down genes in (A). (C) The network plot of enriched terms using cytoscape. Each node represents an enriched term. (D) The cnetplot of representative genes from terms in (A). (E) Genome browser view of scRNA‐seq coverage in the 3′ UTRs of SELENOK, HIF1A, and FCER1G from inflammatory mac and resident mac.

Given that macrophage polarisation involves extensive transcriptomic remodelling, we next asked whether APA contributes to the functional divergence of macrophage subsets. To systematically quantify APA dynamics at single‐cell resolution, we applied the Sierra algorithm to the macrophage scRNA‐seq data (Figure 2A). Sierra enables poly(A) peak detection and UMI‐based quantification across defined clusters, thus allowing precise identification of cell type specific or state dependent APA events, an ideal strategy to resolve post‐transcriptional regulation within macrophage subsets [37]. A global comparison revealed extensive APA reprogramming in the Inflammatory Mac subset, characterised by a marked shift toward proximal PAS usage, indicative of widespread 3′UTR shortening (Figure 4A, S4A). This proximal PAS bias aligns with a transcriptionally hyperactive, metabolically reprogrammed state typical of M1‐like macrophages under inflammatory stimulation. Gene ontology enrichment analysis of genes exhibiting shortened 3′UTRs highlighted functional categories directly related to ‘cytokine production’, ‘lymphocyte activation’, and ‘inflammatory response’ (Figure 4B,C), suggesting that APA remodelling preferentially targets genes critical for immune activation and antibacterial defence.

Notably, key inflammatory regulators such as *HIF1A*, *CXCL8*, *NFKBIZ*, and *SELENOK* displayed pronounced 3′UTR shortening specifically in Inflammatory Mac (Figure 4D,E). Given that 3′UTR shortening can eliminate miRNA or RBP binding sites, these events likely attenuate post‐transcriptional repression, thereby stabilising and amplifying pro‐inflammatory transcripts. In contrast, resident mac cells exhibited an opposite trend toward distal PAS usage and 3′UTR lengthening, with enriched pathways related to ‘cell cycle regulation’ and ‘cytoplasmic translation’, reflecting a state of immune quiescence and tissue maintenance (Figure S4B). Representative genes such as *EREG*, *MXD1*, and *PDE4DIP* exemplify this restraint mechanism (Figure S4C), maintaining longer 3′UTRs to preserve miRNA‐mediated control and prevent excessive activation.

Collectively, these findings provide the first molecular evidence of a post‐transcriptional ‘runaway’ signature in M1‐like macrophages: global APA remodelling drives 3′UTR shortening in inflammation genes, thereby lifting inhibitory constraints and reinforcing pro‐inflammatory activation under chronic bacterial challenge. This APA‐mediated loss of transcriptional braking represents a pivotal molecular mechanism underlying the persistence of destructive inflammation in periodontitis.

### 2.5 *P. gingivalis* Stimulation Instructs Pathogenic APA Remodelling in Macrophages

**FIGURE 5:**
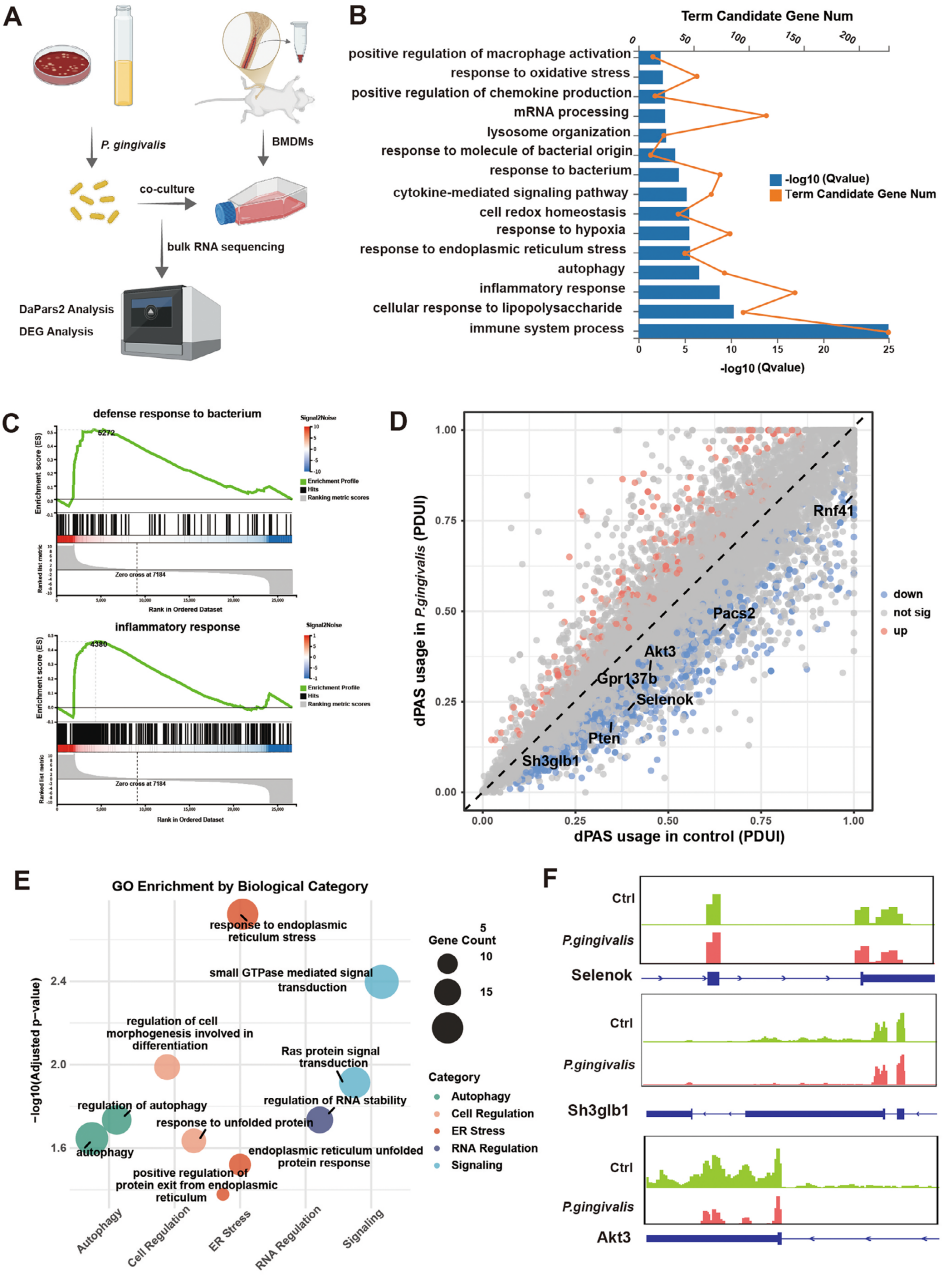
*P. gingivalis* stimulation instructs pathogenic APA remodelling in macrophages. (A) Schematic diagram illustrating bulk RNA sequencing of *P. gingivalis* stimulated BMDMs. (B) GO enrichment plot of upregulated differentially expressed genes in *P. gingivalis* group. *Q*_value_ ≤ 0.05. (C) GSEA analysis plot of upregulated differentially expressed genes in *P. gingivalis* group. (D) Scatter plot of distal polyadenylation site usage index (PDUI) in control and *P. gingivalis* group, analysed using DaPars2. Significant shortening and lengthening for each comparison are highlighted in blue and red, respectively. *p* ≤ 0.05. |ΔPDUI| > 0.25. (E) GO enrichment plot of shortening genes identified in (B). (F) Genome browser view of RNA‐seq coverage in the 3′ UTRs of control and *P. gingivalis* group.

To validate the disease‐associated APA remodelling observed in vivo, we next established an in vitro model of macrophage activation by stimulating BMDMs, corresponding to the monocyte‐derived Inflammatory Mac subset, with *Porphyromonas gingivalis* , a keystone periodontal pathogen (Figure 5A). Transcriptomic profiling confirmed robust macrophage activation: *P. gingivalis* exposure upregulated genes enriched in ‘response to endoplasmic reticulum stress’, ‘autophagy’, ‘response to bacterium’, and ‘inflammatory response‘ (Figure 5B,C), recapitulating the inflammatory program seen in patient‐derived macrophages.

To assess whether pathogen stimulation triggers parallel APA remodelling, we applied the DaPars2 algorithm (dynamic analysis of alternative polyadenylation from RNA‐seq) to quantify global 3′UTR dynamics from bulk RNA‐seq data. APA changes were summarised as the Percentage of distal poly(A) site usage index (PDUI), enabling quantitative comparison of distal versus proximal PAS selection (Figure 5D). Strikingly, *P. gingivalis* stimulation induced a global shift toward proximal PAS usage, indicative of widespread 3′UTR shortening and consistent with a transcriptionally hyperactive macrophage state. Genes exhibiting significant 3′UTR shortening, including *Selenok*, *Sh3glb1*, *Akt3*, and *Gpr137b*, encode proteins involved in stress adaptation, vesicular trafficking, autophagy, and signal transduction, processes tightly linked to macrophage activation and antibacterial defence. Functional enrichment analysis of shortened transcripts revealed strong associations with ‘response to endoplasmic reticulum stress’, ‘regulation of cell morphogenesis involved in differentiation’, ‘autophagy’, and ‘Ras protein signal transduction’ (Figure 5E). These functional categories collectively converge on cellular programs that reinforce inflammatory signalling, cytokine production, and metabolic reconfiguration, hallmarks of M1‐like polarisation [38]. Genome browser visualisation (Figure 5F) confirmed preferential proximal PAS usage in representative targets (*Selenok*, *Sh3glb1*, *Akt3*), validating the APA shortening at the sequence level. Importantly, many of these genes were both transcriptionally upregulated and 3′UTR‐shortened, suggesting that APA remodelling facilitates their expression by relieving miRNA‐ or RBP‐mediated repression.

Together, these findings provide direct evidence that pathogen‐induced macrophage activation is coupled to coordinated APA reprogramming, forming a post‐transcriptional mechanism by which *P. gingivalis* drives runaway M1‐like polarisation. This establishes APA shortening not merely as a correlative signature but as a pathogen‐instructed molecular event that reinforces inflammatory gene expression in periodontitis.

### 2.6 APA‐Mediated 3′UTR Shortening of *Selenok* Severs the miR‐320‐3p ‘Brake Line’

**FIGURE 6:**
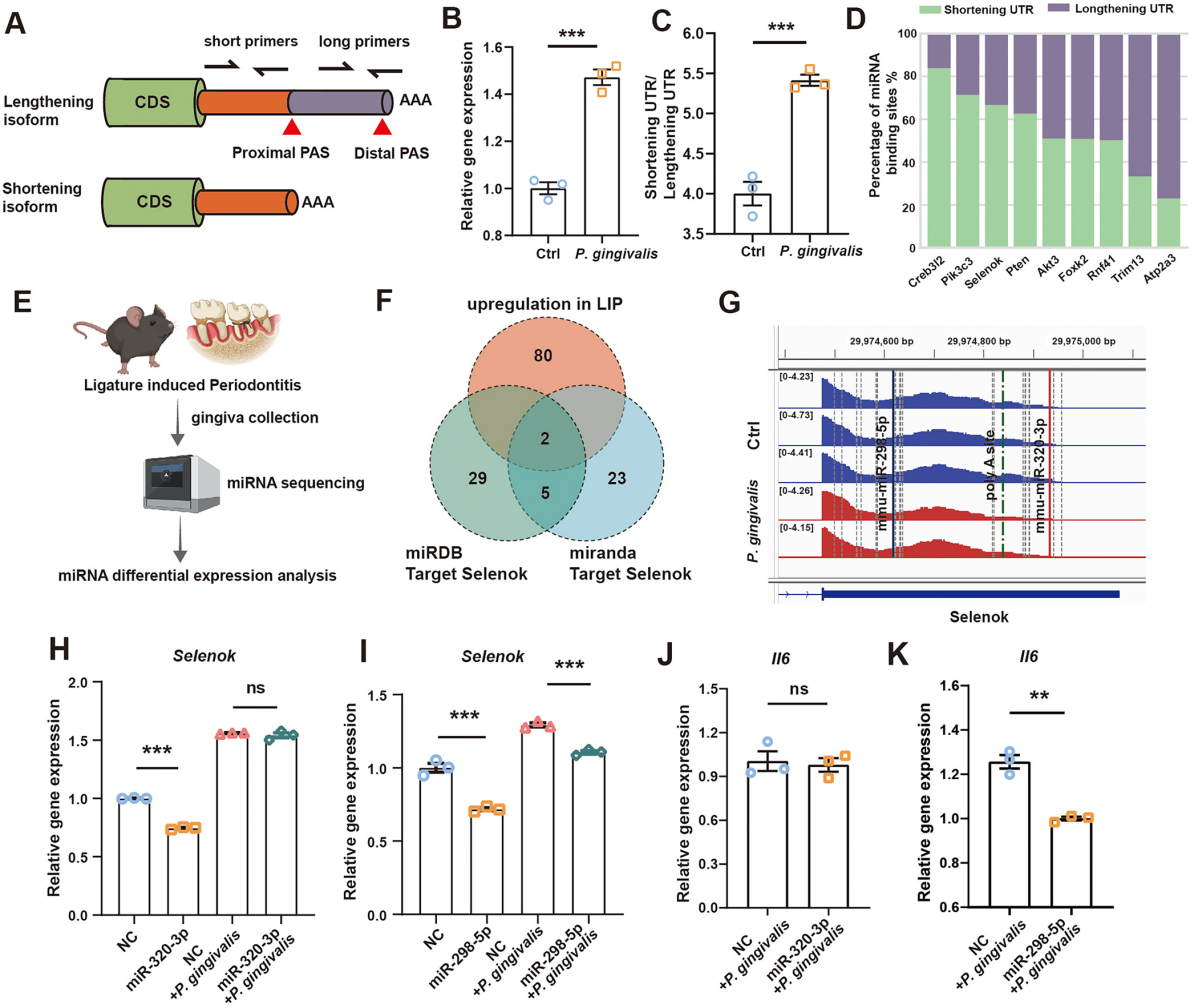
APA‐mediated 3′UTR shortening of *Selenok* severs the miR‐320‐3p ‘brake line’. (A) Schematic illustration showing the design of isoform‐specific primers targeting the long and short 3′UTR regions of *Selenok*. (B) qRT‐PCR analysis of *Selenok* mRNA expression in control and *P. gingivalis* stimulated macrophages. (C) Ratio of shortening/lengthening 3′UTR isoforms of *Selenok* in control and *P. gingivalis* groups. (D) Bar plot showing the percentage of predicted miRNA‐binding sites in transcripts with shortened versus lengthening 3′UTRs. (E) Schematic overview of small RNA sequencing performed on gingival tissues from experimental periodontitis and control mice. (F) Venn diagram illustrating the overlap between upregulated miRNAs in ligature‐induced periodontitis (LIP) and *Selenok*‐targeting miRNAs predicted by miRDB and miRanda. (G) Genome browser view displaying the positions of miR‐298‐5p, miR‐320‐3p, and the poly(A) site within the *Selenok* 3′UTR. (H) qRT‐PCR quantification of *Selenok* expression across four experimental groups following miR‐320‐3p manipulation. (I) qRT‐PCR quantification of *Selenok* expression across four experimental groups following miR‐298‐5p manipulation. (J) qRT‐PCR quantification of *Il6* expression following miR‐320‐3p manipulation and *P. gingivalis* infection. (K) qRT‐PCR quantification of *Selenok* expression across four experimental groups following miR‐320‐3p manipulation. *p < 0.05, **p < 0.01, and ***p < 0.001.

To mechanistically link APA remodelling with the release of post‐transcriptional inhibition, we focused on *Selenok*, an antioxidant and immune‐regulatory gene that exhibited a pronounced proximal APA shift both in vivo (inflammatory mac) and in vitro ( *P. gingivalis* –stimulated BMDMs). *Selenok* was selected as a representative example because it integrates redox regulation and inflammatory signalling, two processes tightly coupled to macrophage hyperactivation [39, 40, 41]. *Selenok* generates two major transcript isoforms through alternative usage of proximal and distal poly(A) sites, as illustrated schematically and validated using isoform‐specific primers (Figure 6A). *Selenok* was significantly upregulated in inflammatory macrophages and following *P. gingivalis* stimulation, as demonstrated by violin plots and RT‐qPCR (Figures 6B, S5A,B). It was accompanied by a significant elevation in the short/long 3′UTR isoform ratio (Figure 6C), indicating activation of proximal APA under bacterial challenge.

To assess the regulatory impact of 3′UTR shortening, we performed a genome‐wide analysis of miRNA‐binding site loss across APA‐regulated genes. The degree of 3′UTR shortening strongly correlated with the number of lost miRNA sites (*r* = 0.77, *p* < 2.2e^−16^; Figure S5C), establishing APA as a major determinant of miRNA repression potential. *Selenok* ranked among the top genes showing extensive miRNA‐site loss (Figure 6D). To identify disease‐relevant miRNAs potentially involved in this regulation, we performed miRNA‐seq on gingival tissues from experimental periodontitis and control mice (Figure 6E). Intersection of upregulated miRNAs with *Selenok*‐targeting predictions identified miR‐298‐5p and miR‐320‐3p as candidate regulators (Figure 6F and Table S1). Sequence alignment revealed that the miR‐320‐3p recognition motif resides exclusively within the distal 3’UTR region removed by proximal APA, whereas the miR‐298‐5p site remains intact in the shortened isoform (Figure 6G). Dual‐luciferase reporter assays confirmed direct binding of both miRNAs to their predicted *Selenok* 3UTR target sites (Figure S5D,E).

Functionally, transfection of miRNA mimics in BMDMs revealed that miR‐320‐3p strongly repressed *Selenok* under basal conditions, yet this suppression was completely abolished following *P. gingivalis* stimulation (Figure 6H), when *Selenok* predominantly existed as the short 3′UTR isoform lacking the miR‐320‐3p site. In contrast, miR‐298‐5p retained partial inhibitory activity (Figure 6I), consistent with preservation of its binding motif. This derepression of Selenok was functionally reflected in cytokine output, as miR‐298‐5p overexpression led to a marked reduction in IL6 production, whereas miR‐320‐3p had no significant effect, confirming the selective post‐transcriptional regulation of inflammatory activation (Figure 6J,K).

Collectively, these findings delineate a precise molecular mechanism of ‘brake failure’ in macrophage inflammatory control. In the periodontitis microenvironment, the host attempts to restrain excessive inflammation by upregulating miR‐320‐3p, which normally acts as a post‐transcriptional brake on *Selenok* expression. However, *P. gingivalis* , induced APA remodelling shortens the *Selenok* 3′UTR, effectively cutting this ‘brake line’ by removing the miR‐320‐3p binding site. As a result, miRNA repression is abolished, allowing *Selenok* and its downstream inflammatory effectors to escape control and sustain a hyperactivated M1‐like state. Notably, miRNAs such as miR‐298‐5p, whose target sites remain unaffected by APA, may still exert partial repression, providing a potential compensatory route to restore post‐transcriptional restraint and rebalance macrophage activation.

## 3 Discussion

This study reveals APA as a critical post‐transcriptional mechanism that drives pathological M1 macrophage polarisation in periodontitis. While previous studies have centred on transcriptional activation as the primary driver of macrophage function [42, 43, 44, 45], our findings address a key pathological paradox: how pro‐inflammatory macrophage programs are relentlessly sustained despite the presence of endogenous inhibitory mechanisms. We demonstrate that in the chronic infectious microenvironment of periodontitis, MDMs undergo a global, adaptive shift toward proximal PAS usage. This 3′UTR shortening is not merely an epiphenomenon of activation but a distinct pathogenic strategy that ‘derepresses’ the M1 inflammatory program, enabling it to become ‘runaway’ and uncontrolled. By integrating single cell transcriptomics with the Sierra APA framework, we uncovered the layer of isoform level dysregulation, which remains invisible to conventional analyses focused solely on gene expression intensity or kinetic trajectories. Sierra analyzes transcriptomic features at the single cell level, effectively leveraging thousands of cells per sample [29]. As a result, even with three biological replicates per group, the analysis captures sufficient cellular heterogeneity to draw robust conclusions, and the limited number of samples has minimal impact on the reliability of the findings.

Our data propose a model wherein chronic inflammation is sustained by a pathogenic ‘disruption’ of post‐transcriptional control. A healthy immune response requires negative feedback loops, such as miRNA‐mediated repression [46, 47], to act as ‘brakes’ and prevent excessive inflammation [48, 49]. Our study resolves the apparent failure of these brakes in periodontitis by identifying APA as the ‘disruption’ mechanism. We provide evidence that the widespread 3′UTR shortening observed in Inflammatory Mac correlates directly with a significant loss of miRNA binding sites. Crucially, this APA‐mediated ‘rewiring’ is not random; it selectively targets genes enriched in ‘cytokine production’, ‘lymphocyte activation’, and ‘inflammatory signalling pathway’. This implies a coordinated post‐transcriptional strategy to uncouple the entire M1‐like inflammatory engine from its key inhibitory brakes, thereby locking the macrophage in a hyper‐activated, tissue‐destructive state.

The *Selenok*/miR‐320‐3p axis provides a precise, mechanistic ‘snapshot’ of this ‘brake‐cutting’ process. *Selenok* was identified as a top‐ranking gene undergoing robust APA‐mediated 3′UTR shortening in both in vivo Inflammatory Mac and in vitro *P. gingivalis* ‐stimulated BMDMs. Our small RNA sequencing of diseased gingival tissues confirmed that the host, as expected, attempts to apply the brakes by upregulating inhibitory miRNAs, including miR‐320‐3p. Herein lies the ‘hijacking’: the *P. gingivalis* ‐induced APA shift generating the short *Selenok* isoform selectively excises the binding site for miR‐320‐3p. Our functional validation unequivocally demonstrates the consequence: the inhibitory power of miR‐320‐3p, which is effective under basal conditions, is completely abolished in *P. gingivalis* ‐stimulated macrophages. This finding provides definitive, proof‐of‐concept evidence for how pathogen‐induced APA remodelling directly facilitates escape from miRNA‐mediated suppression.

This selective ‘editing’ of the regulatory interface is a key insight. The 3′UTR shortening of *Selenok* did not remove the binding site for miR‐298‐5p, which retained partial regulatory activity. This demonstrates that APA acts as a highly specific ‘rheostat’ or ‘dimmer switch’ rather than a simple ‘on/off’ toggle. It does not abolish miRNA control globally but precisely sculpts the 3′UTR landscape to ‘tune out’ specific inhibitory signals (miR‐320‐3p) while preserving others. While *Selenok* serves as a powerful proof of concept, our data strongly suggest this is a global phenomenon. The concurrent 3′UTR shortening of other master regulators of the M1 phenotype, such as *HIF1A* (metabolic reprogramming), *CXCL8* (neutrophil recruitment), and *NFKBIZ* (NF‐κB signalling), indicates a systemic, coordinated ‘derepression’ of the M1 machinery [50, 51, 52]. This global escape from post‐transcriptional suppression provides a compelling molecular rationale for the chronic, non‐resolving inflammation characteristic of periodontitis.

To mechanistically interrogate the APA remodelling identified in human gingival macrophages, we employed *P. gingivalis* stimulated murine BMDMs as an experimentally tractable model system. The fundamental logic of APA regulation, including CPSF/CSTF/CFIm‐CFIIm mediated poly(A) site recognition, RNA polymerase II elongation coupled PAS selection, and stress induced shifts toward proximal PAS usage, relies on conserved biochemical mechanisms across mammalian macrophages [19, 35]. Consistently, *Selenok* displays a comparable dual‐isoform 3′UTR architecture in both species, and inflammatory activation similarly biases its PAS choice toward the proximal site, supporting the suitability of the murine system for probing causal links between APA remodelling and inflammatory polarisation. We recognise that certain quantitative differences such as species dependent 3′UTR lengths and miRNA target site composition may modulate the quantitative extent of APA shifts and the relative preference for specific poly(A) sites. Furthermore, BMDM‐ *P. gingivalis* interactions represent a reductionist macrophage‐pathogen axis and cannot fully reproduce the complex periodontal milieu shaped by multispecies biofilms, hypoxia, extracellular matrix remodelling, and stromal‐immune crosstalk. These context dependent factors may further tune APA outcomes in vivo but do not alter the mechanistic principles elucidated here.

The use of *P. gingivalis* was motivated by its unique ability, as the keystone periodontal pathogen, to engage multiple pattern‐recognition and metabolic pathways simultaneously through atypical lipid A structures, gingipain proteases, and coordinated TLR2/TLR4 signalling [2, 27]. Such multifactorial activation more faithfully recapitulates the integrated upstream cues that converge on APA regulation, whereas single‐component stimuli such as purified LPS lack the signalling complexity required to model APA remodelling. Although simplified, this controlled system provides a biologically relevant platform for dissecting pathogen‐induced APA mechanisms that are otherwise inaccessible in primary human gingival macrophages.

From a therapeutic perspective, this study shifts the focus from merely blocking inflammation to restoring immune regulation. The identification of the APA‐miRNA axis as a key node of pathogenic control suggests a novel strategy: repairing the brakes. Rather than using broad‐spectrum anti‐inflammatory drugs, which can compromise host defence, it may be possible to therapeutically target the APA machinery itself (e.g., core factors like NUDT21 or CFIm) [53, 54, 55]. Such an approach could force pro‐inflammatory transcripts back into their long‐3′UTR isoforms, thereby re‐sensitising them to the host's endogenous miRNA brakes and recalibrating the runaway M1 response. While broader experimental validation across additional APA‐regulated genes is essential, our findings establish APA remodelling as a critical, druggable interface in the host‐pathogen battle, offering a new paradigm for treating macrophage‐driven chronic inflammatory diseases.

## 4 Conclusion

In summary, this study identifies APA as a key pathogenic mechanism driving the runaway M1 macrophage polarisation that characterises periodontitis. We demonstrate that chronic bacterial challenge induces a global shift toward proximal PAS usage in pro‐inflammatory macrophages, resulting in widespread 3′UTR shortening. This APA‐mediated remodelling constitutes a form of post‐transcriptional disruption, systematically excising inhibitory miRNA‐binding sites from critical inflammatory genes. As mechanistically demonstrated by the *Selenok* gene, this 3′UTR shortening ‘cuts the brake lines’ by selectively abolishing repression from host‐induced miRNAs like miR‐320‐3p. This derepression of the M1 inflammatory machinery explains the failure of endogenous immune brakes, leading to the uncontrolled, self‐sustaining inflammation that drives tissue destruction. Our findings establish the APA–miRNA axis as a critical interface of host‐pathogen conflict, offering a new therapeutic paradigm for chronic inflammatory diseases focused on restoring, rather than merely suppressing, immune regulation.

## 5 Methods

### 5.1 Ethics Statement

The study protocol involving human participants was rigorously reviewed and formally approved by The Ethics Committee of School & Hospital of Stomatology, Wuhan University (WDKQ2025). All procedures were conducted in strict accordance with the ethical principles outlined in the Declaration of Helsinki. Prior to sample collection, all participants were provided with a detailed explanation of the study's objectives and procedures, and written informed consent was obtained from each individual. All animal experiments were designed and performed in compliance with the ARRIVE guidelines and were approved by the Animal Care and Use Committee of the Medical Research Institute, Wuhan University (MLIC2021175), ensuring the humane treatment and welfare of the animals throughout the study.

### 5.2 Human Gingival Tissue Collection and Single‐Cell Suspension Preparation

Gingival tissue biopsies were obtained from three patients diagnosed with chronic periodontitis and three periodontally healthy control subjects undergoing routine dental procedures. The diagnosis of periodontitis was established based on a comprehensive clinical examination, including criteria such as probing depth (PD) ≥ 5 mm, clinical attachment loss (CAL) ≥ 3 mm, and radiographic evidence of alveolar bone loss. Healthy controls exhibited no signs of periodontal inflammation, with PD ≤ 3 mm and no CAL. After being harvested, tissues were washed in ice‐cold PBS (Hyclone, SH30256.01) and dissociated using Collagenase II (Sigma, V900892). DNase I (Sigma, 9003‐98‐9) treatment was applied as needed depending on the viscosity of the homogenate. Following erythrocyte removal (Solarbio, R1010), cell count and viability were estimated using a Fluorescence Cell Analyser (Countstar Rigel S2) with AO/PI reagent. Debris and dead cells were removed if necessary (Miltenyi, 130‐109‐398/130‐090‐101). Finally, fresh cells were washed twice in RPMI 1640 (Gibco, 11875119) and then resuspended at 1 × 10^6^ cells/mL in RPMI 1640 supplemented with 2% FBS (Gibco, 10100147C) for subsequent analysis.

### 5.3 Single‐Cell RNA Sequencing Library Construction and Sequencing

Single‐cell RNA‐Seq libraries were prepared using the SeekOne MM Single Cell 3′ library preparation kit (SeekGene, K00104). An appropriate number of cells were loaded into the microwells of a SeekOne MM chip. After the cells settled by gravity, cell barcoded magnetic beads (CBBs) were added and allowed to settle in the microwells with the aid of a magnetic field. Excess beads were removed, and the cells were lysed, allowing their RNA to be captured by the CBBs in the same microwell. The CBBs were then collected, and reverse transcription was performed to label the cDNA with cell‐specific barcodes. Unused primers were removed with Exonuclease I treatment. Subsequently, the barcoded cDNA on the CBBs was used as a template for second‐strand synthesis. The resulting double‐stranded DNA was denatured off the CBBs, purified, and amplified. The amplified cDNA was then used to construct the final sequencing libraries, which included full‐length sequencing adapters and sample indices. The indexed libraries were purified with VAHTS DNA Clean Beads (Vazyme, N411‐01), quantified using a Qubit fluorometer (Thermo Fisher Scientific, Q33226), and their fragment size distribution was analysed with a Bio‐Fragment Analyser (Bioptic, Qsep400). The libraries were sequenced on an Illumina NovaSeq 6000 platform with a PE150 read length.

### 5.4 scRNA‐Seq Data Processing and Analysis

Raw sequencing data (FASTQ format) were processed using the seeksoultools software pipeline (version 1.3.0). Reads were aligned to the human reference genome (GRCh38) to generate a feature‐barcode matrix. Downstream analysis was conducted using the Seurat R package (version 5.3.0). Stringent quality control was applied to filter out low‐quality cells and potential doublets, excluding cells with fewer than 200 or more than 6000 detected genes, or with total counts more than 40,000 per cell, or with a mitochondrial gene percentage greater than 20%. The filtered data were then normalised using the LogNormalize method. To correct for batch effects, the RPCA (reciprocal principal component analysis) integration method was utilised. Highly variable genes were identified to capture biological heterogeneity while reducing computational noise. Principal component analysis (PCA) was performed for linear dimensionality reduction, and the optimal number (n_pcs = 13) of principal components was selected for downstream clustering and visualisation using uniform manifold approximation and projection (UMAP). For macrophage‐specific analysis, the macrophage population was computationally isolated and re‐clustered. Differentially expressed genes (DEGs) between clusters were identified using the Wilcoxon rank‐sum test, with a significance threshold of adjusted *p*‐value < 0.05, an absolute log_2_ (fold change) > 0.25 and a min expression percent of cell > 0.25.

### 5.5 Pseudotime Trajectory and RNA Velocity Analysis

To reconstruct the developmental relationships and differentiation trajectories of macrophage subsets, pseudotime analysis was performed using the Monocle 3 R package (version 1.3.7). To further investigate the directionality and kinetics of these transitions, RNA velocity analysis was conducted using the velocyto (version 0.17.16) and scVelo toolkit (version 0.3.3) in Python. The resulting velocity vectors were embedded and visualised on the UMAP projection to illustrate the directional flow of macrophage differentiation.

### 5.6 Single‐Cell Alternative Polyadenylation (APA) Analysis

To profile APA dynamics from the 3′ biased scRNA‐seq data, we employed the Sierra algorithm (version 0.99.27). We performed de novo poly(A) peak calling within defined cell clusters and quantified the usage of each peak with UMIs using the Sierra algorithm, which robustly identified cell‐type‐specific shifts in poly(A) site selection. To identify genes undergoing significant APA remodelling, we compared the relative usage of proximal versus distal poly(A) sites between macrophage subsets using the DEXSeq package, applying a significance threshold of an adjusted *p*‐value (padj) < 0.05.

### 5.7 Animals and Experimental Periodontitis Model

Six to eight‐week‐old female C57BL/6 mice were used for the in vivo experiments. The animals were housed under specific pathogen‐free conditions with a 12‐h light/dark cycle and ad libitum access to food and water. To induce experimental periodontitis, mice were anaesthetised, and a 5‐0 silk ligature was carefully placed around the maxillary second molar to facilitate subgingival plaque accumulation and trigger an inflammatory response. The ligatures remained in place for 4 days. Control mice underwent a sham procedure without ligature placement. There were four mice each in the periodontitis group and the control group. At the end of the experimental period, gingival tissues surrounding the molars were harvested for subsequent small RNA sequencing.

### 5.8 Cell Culture and *P. gingivalis* Stimulation

Bone marrow‐derived macrophages (BMDMs) were generated by flushing bone marrow from the femurs and tibias of six‐week‐old female C57BL/6 mice. The progenitor cells were cultured for 7 days in Dulbecco's modified eagle medium (DMEM) supplemented with 10% fetal bovine serum (FBS), 1% penicillin–streptomycin, and 20 ng/mL macrophage colony‐stimulating factor (M‐CSF) to promote differentiation into macrophages. The keystone periodontal pathogen *Porphyromonas gingivalis* (ATCC 33277) was cultured in brain heart infusion (BHI) broth supplemented with 5 μg/mL hemin and 1 μg/mL Vitamin K1, and maintained under strict anaerobic conditions in an atmosphere of 80% N_2_, 10% H_2_, and 10% CO_2_ at 37°C. For stimulation experiments, differentiated BMDMs were challenged with live *P. gingivalis* at a multiplicity of infection (MOI) of 100 for 12 h to mimic the chronic bacterial challenge observed in periodontitis.

### 5.9 Bulk RNA Sequencing and APA Analysis

For bulk RNA sequencing, BMDMs were stimulated with *P. gingivalis* at an MOI of 100 for 12 h. Total RNA was subsequently isolated using TRIzol reagent. The construction of cDNA libraries and sequencing were performed on the BGISEQ200 platform (BGI, Shenzhen, China). The resulting clean data were initially analysed and mapped using the Dr. Tom platform. Differential gene expression between the stimulated and control groups was determined using the DESeq2 package (v1.4.5), with a *Q*‐value threshold of ≤ 0.05. To specifically investigate alternative polyadenylation, we utilised the DaPars2 (version 2.1) algorithm on the sequencing data. This approach identified de novo poly(A) sites and calculated the percentage of distal polyA site usage index (PDUI) for each gene. A significant shift toward proximal poly(A) site usage, indicative of 3′ UTR shortening, was defined as a substantial decrease in PDUI (ΔPDUI < −0.25 and *p*‐value ≤ 0.05).

### 5.10 Small RNA Sequencing and Analysis

Total RNA was extracted from gingival tissues of experimental mice for small RNA analysis. Sequencing libraries were generated using the NEBNext Multiplex Small RNA Library Prep Set for Illumina (NEB, E7300L). The procedure involved ligating 3′ and 5′ adaptors, followed by reverse transcription to cDNA and PCR amplification. Libraries with insert sizes of 18–40 bp were selected and sequenced on an Illumina NovaSeq 6000 platform with a single‐end 50 bp strategy at Novogene Co. Ltd. For bioinformatic analysis, raw reads were filtered to obtain clean reads, which were then mapped to the mouse reference genome using Bowtie. Known miRNAs were identified by aligning reads to the miRBase database (version 20.0) with mirdeep2 (version 0.1.3). miRNA expression levels were quantified as transcripts per million (TPM), and differential expression analysis between groups was performed using the DESeq R package (version 1.49.7).

### 5.11 miRNA Target Prediction and Functional Enrichment Analysis

To identify potential miRNA‐target interactions, we used a combinatorial approach. The 3′ UTR sequences of genes of interest were scanned for potential miRNA binding sites using two independent prediction algorithms, miRanda (version 3.3) and miRDB (version 6.0). To interpret the biological significance of large gene sets (DEGs or genes with APA shifts), gene ontology (GO) enrichment analysis was performed. The results, including network plots of functionally related enriched terms, were visualised using Cytoscape (version 3.10.3) to provide a systems‐level view of the regulatory landscape.

### 5.12 miRNA Mimic Transfection

To functionally validate the inhibitory effect of specific miRNAs on target gene expression, synthetic miRNA mimics (miR‐298‐5p, miR‐320‐3p) and a non‐targeting negative control (NC) mimic were purchased from GenePharma. Differentiated BMDMs were transfected with 50 nM of the miRNA mimics using Lipofectamine 3000 transfection reagent according to the manufacturer's protocol. 12 h post‐transfection, cells were either left unstimulated or were stimulated with *P. gingivalis* for an additional 12 h before being harvested for gene expression analysis.

### 5.13 RNA Extraction and Quantitative Real‐Time PCR (qRT‐PCR)

Total RNA was extracted from cultured BMDMs using RNAiso Plus Total RNA extraction reagent (TaKaRa, Cat. #9109). The concentration and purity of the extracted RNA were assessed using a NanoDrop spectrophotometer. For mRNA quantification, 1 μg of total RNA was reverse‐transcribed into cDNA using ABScript III RT Master Mix for qPCR with gDNA Remover (ABclonal, RK20429). Quantitative real‐time PCR was subsequently performed in triplicate on a LightCycler 480 system using Universal SYBR Green Fast qPCR Mix (ABclonal, RK21203). The relative expression levels of target mRNAs were normalised to the endogenous control GAPDH and calculated using the 2^−ΔΔCt^ method. The primer sequences used were as follows: Selenok CDS region (F: 5′‐CTTTCCTAACAGACTTCTTCTGGG‐3′, R: 5′‐CCTCTTCCATCGTCGTATCTGG‐3′); Selenok short 3′UTR (F: 5′‐CATCAAGCGGTGAAGAGCGG‐3′, R: 5′‐GGCTAGATCCTGCAGAGGGAA‐3′); Selenok long 3′UTR (F: 5′‐ATCACCCCCTTCTCCAGTGTA‐3′, R: 5′‐AGAGCAAAGTAACAGTGAGCAGTA‐3′); and Gapdh (F: 5′‐AGGTCGGTGTGAACGGATTTG‐3′, R: 5′‐TGTAGACCATGTAGTTGAGGTCA‐3′).

### 5.14 Statistical Analysis

All statistical analyses for in vitro experimental data were performed using GraphPad Prism (version 8.0.2). Data are presented as the mean ± standard error of the mean (SEM) from at least three independent experiments. Statistical comparisons between two groups were made using a two‐tailed student's *t*‐test. A *p*‐value of less than 0.05 was considered statistically significant. Significance levels in figures are denoted as follows: *p < 0.05, **p < 0.01, and ***p < 0.001.

## Author Contributions

Jing Zhang and Yilong Zhao contributed to design, acquisition, and analysis, drafted and critically revised the manuscript. Jiaru Deng contributed to acquisition and analysis, and critically revised the manuscript. Shuyuan Qu and Yiyi Zhou contributed to acquisition and critically revised the manuscript. Qin Zhao contributed to conception and design, contributed to acquisition, drafted, and critically revised the manuscript. Yufeng Zhang contributed to analysis and interpretation, and critically revised the manuscript.

## Funding

This work was supported by the National Natural Science Foundation of China (82530028, 82220108018, 82270981, 82571092), the Natural Science Foundation of Hubei Province, China (2025AFA082), the Fundamental Research Funds for the Central Universities (2042025YXB013), and the Interdisciplinary Research Project of School of Stomatology Wuhan University (XNJC202302).

## Conflicts of Interest

The authors declare no conflicts of interest.

## Supporting information


**Figure S1:** (A) UMAP plot of all cell clusters. (B) Heatmap displaying the top 10 differentially expressed marker genes across all cell populations. (C) UMAP plot of all cell populations in the health and periodontitis groups. (D) Cell proportion of all cell populations in the healthy and periodontitis groups. (E) UMAP plot of representative marker gene expression in the immune cell populations. (F) Heatmap displaying the interactions among all cell populations. (G) Dot plot showing the incoming and outgoing interaction strength among all cell populations. (H) Il6 signalling pathway network in all cell populations.
**Figure S2:** (A) Bar plots displaying the percentage of macrophage subsets in the healthy and periodontitis groups. (B) GO enrichment of down‐regulated genes in Figure 2E.
**Figure S3:** (A) Scatter plot of *S100A8* RNA velocity showing the relationship between unspliced and spliced RNA transcripts across different macrophage subsets. (B) UMAP plot of RNA velocity (left) and gene expression (right) of *S100A8*. (C) Scatter plot of *FOLR2* RNA velocity showing the relationship between unspliced and spliced RNA transcripts across different macrophage subsets. The dashed line indicates the expected steady‐state ratio. (D) UMAP plot of RNA velocity (left) and gene expression (right) of *FOLR2*.
**Figure S4:** (A) Counts of 3′UTR peaks showing differential usage according to their relative location to the terminating exon. Location of 0 indicates the peak most proximal to the terminating exon, with 1 representing the most distal. Comparisons performed are for Inflammatory Mac and MHCII^hi^ Mac. (B) GO enrichment plot of up genes in Figure 4A. (C) Genome browser view of scRNA‐seq coverage in the 3′ UTRs of *EREG*, *MXD1* and *PDE4DIP* from inflammatory mac and resident mac.
**Figure S5:** (A) Violin plot of *SELENOK* expression in inflammatory mac versus resident mac. (B) Violin plot of *SELENOK* expression in macrophages: healthy versus periodontitis. (C) Scatter plot displaying 3′UTR length change and miRNA binding sites lost. (D) Bar chart of dual luciferase reporter assay for miRNA 298‐5p. (E) Bar chart of dual luciferase reporter assay for miRNA 320‐3p.
**Table S1:**. Up‐regulation miRNAs identified by small RNA sequencing in periodontitis versus control.

## Data Availability

Bulk RNA sequencing data are available at SRA database under the accession number PRJNA863330. All data supporting the findings of this study are available within the paper and its Supporting Information file.
