# Supplementary material for: Alternative Polyadenylation Drives Runaway Pro‐Inflammatory Macrophages in Periodontitis by Enabling Escape From miRNA Repression
Source: Cell Prolif. 2025 Dec 22;59(6):e70156. doi: 10.1111/cpr.70156 (PMC13241824; doi:10.1111/cpr.70156)
Supplement: Supplementary file 1 — Figure S1: (A) UMAP plot of all cell clusters. (B) Heatmap displaying the top 10 differentially expressed marker genes across all cell populations. (C) UMAP plot of all cell populations in the health and periodontitis groups. (D) Cell proportion of all cell populations in the healthy and periodontitis groups. (E) UMAP plot of representative marker gene expression in the immune cell populations. (F) Heatmap displaying the interactions among all cell populations. (G) Dot plot showing the incoming and outgoing interaction strength among all cell populations. (H) Il6 signalling pathway network in all cell populations. Figure S2: (A) Bar plots displaying the percentage of macrophage subsets in the healthy and periodontitis groups. (B) GO enrichment of down‐regulated genes in Figure 2E. Figure S3: (A) Scatter plot of S100A8 RNA velocity showing the relationship between unspliced and spliced RNA transcripts across different macrophage subsets. (B) UMAP plot of RNA velocity (left) and gene expression (right) of S100A8. (C) Scatter plot of FOLR2 RNA velocity showing the relationship between unspliced and spliced RNA transcripts across different macrophage subsets. The dashed line indicates the expected steady‐state ratio. (D) UMAP plot of RNA velocity (left) and gene expression (right) of FOLR2. Figure S4: (A) Counts of 3′UTR peaks showing differential usage according to their relative location to the terminating exon. Location of 0 indicates the peak most proximal to the terminating exon, with 1 representing the most distal. Comparisons performed are for Inflammatory Mac and MHCIIhi Mac. (B) GO enrichment plot of up genes in Figure 4A. (C) Genome browser view of scRNA‐seq coverage in the 3′ UTRs of EREG, MXD1 and PDE4DIP from inflammatory mac and resident mac. Figure S5: (A) Violin plot of SELENOK expression in inflammatory mac versus resident mac. (B) Violin plot of SELENOK expression in macrophages: healthy versus periodontitis. (C) Scatter plot displaying 3′UTR length ch [file CPR-59-e70156-s001.docx]

**Supplementary Materials**

**Alternative polyadenylation drives runaway pro-inflammatory macrophages**

**in periodontitis by enabling escape from miRNA repression**

Jing Zhang^1, †^, Yilong Zhao^1, †^, Jiaru Deng^1^, Shuyuan Qu^1^, Yiyi Zhou^1^, Qin Zhao^1,^ *, Yufeng Zhang^1,2,3,^ *

^1^State Key Laboratory of Oral & Maxillofacial Reconstruction and Regeneration, Key Laboratory of Oral Biomedicine Ministry of Education, Hubei Key Laboratory of Stomatology, School & Hospital of Stomatology, Wuhan University; Wuhan 430079, China.

^2^Medical Research Institute, School of Medicine, Wuhan University; Wuhan 430071, China

^3^Taikang Center for Life and Medical Sciences, Wuhan University; Wuhan 430071, China

* Corresponding author. Yufeng Zhang, [zyf@whu.edu.cn](mailto:zyf@whu.edu.cn)

Qin Zhao, zhaoqin@whu.edu.cn

† These authors contributed equally.


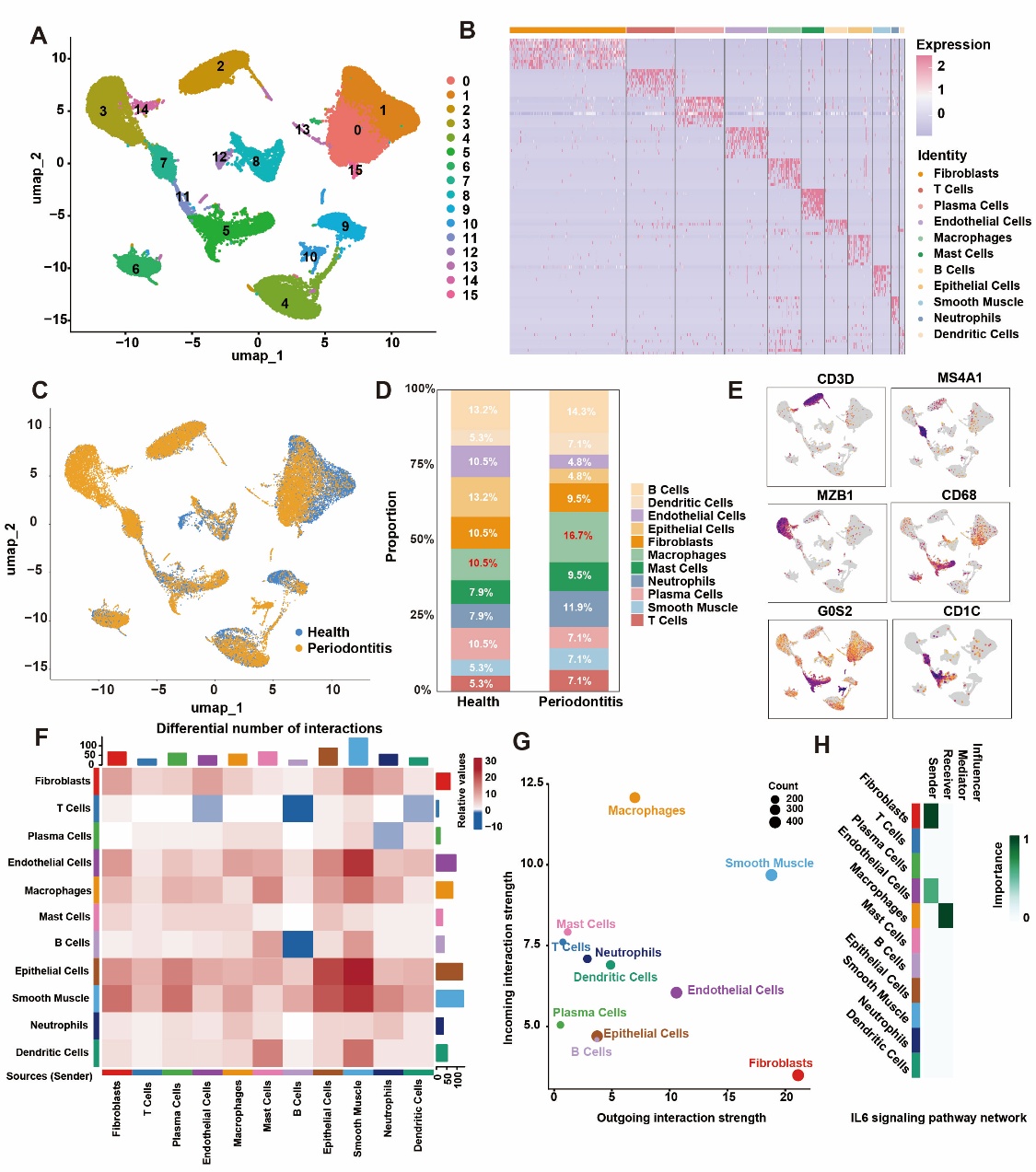


**Figure S1.**

(A) UMAP plot of all cell clusters. (B) Heatmap displaying the top 10 differentially expressed marker genes across all cell populations. (C) UMAP plot of all cell populations in the Health and Periodontitis groups. (D) Cell proportion of all cell populations in the Healthy and Periodontitis groups. (E) UMAP plot of representative marker gene expression in the immune cell populations. (F) Heatmap displaying the interactions among all cell populations. (G) Dot plot showing the incoming and outgoing interaction strength among all cell populations. (H) Il6 signaling pathway network in all cell populations.


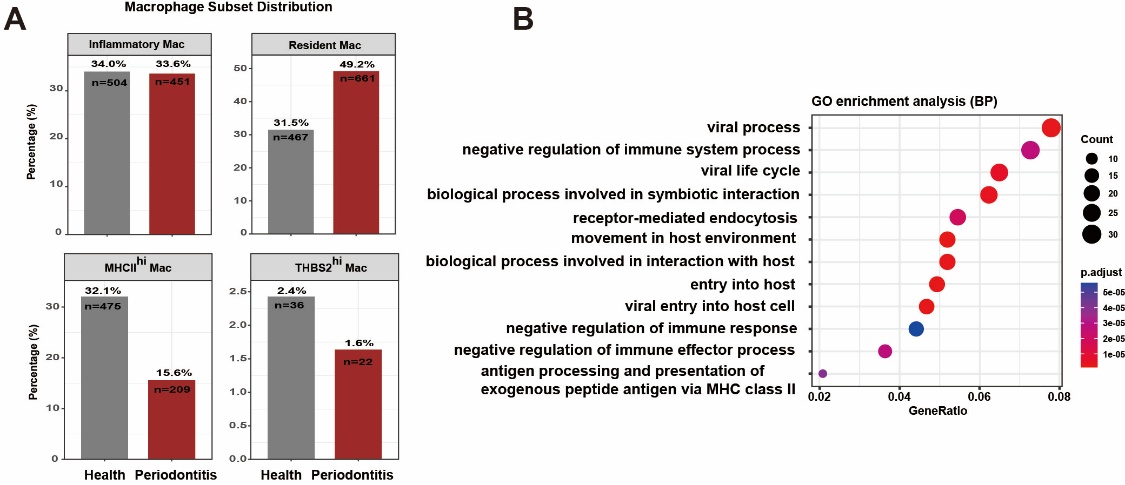


**Figure S2.**

(A) Bar plots displaying the percentage of macrophage subsets in the Healthy and Periodontitis groups. (B) GO enrichment of down-regulated genes in Figure 2E.


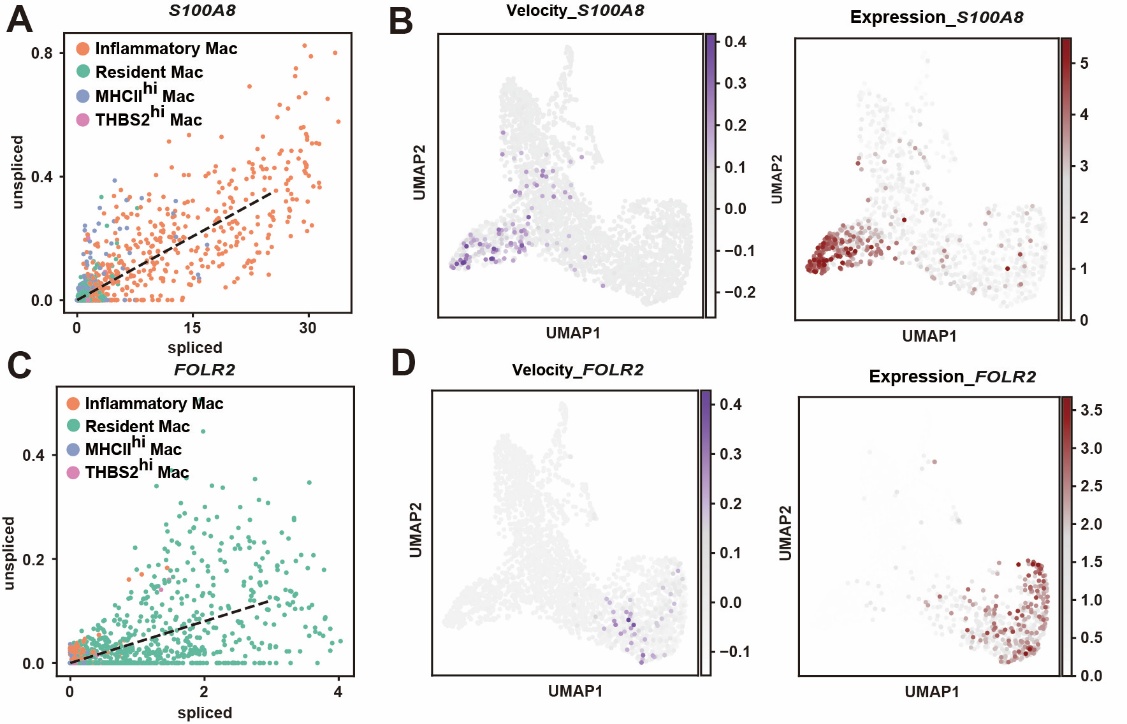


**Figure S3.**

(A) Scatter plot of *S100A8* RNA velocity showing the relationship between unspliced and spliced RNA transcripts across different macrophage subsets. (B) UMAP plot of RNA velocity (left) and gene expression (right) of *S100A8*. (C) Scatter plot of *FOLR2* RNA velocity showing the relationship between unspliced and spliced RNA transcripts across different macrophage subsets. The dashed line indicates the expected steady-state ratio. (D) UMAP plot of RNA velocity (left) and gene expression (right) of *FOLR2*.


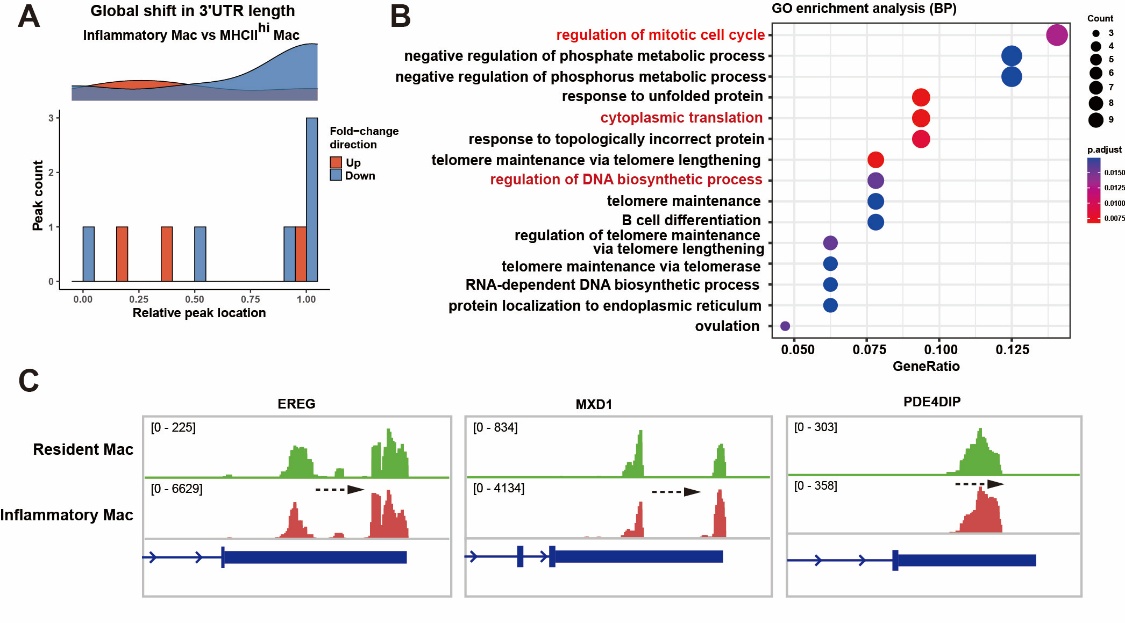


**Figure S4.**

(A) Counts of 3’UTR peaks showing differential usage according to their relative location to the terminating exon. Location of 0 indicates the peak most proximal to the terminating exon, with 1 representing the most distal. Comparisons performed are for Inflammatory Mac and MHCII^hi^ Mac. (B) GO enrichment plot of up genes in Figure 4A. (C) Genome browser view of scRNA-seq coverage in the 3’ UTRs of *EREG*, *MXD1* and *PDE4DIP* from Inflammatory Mac and Resident Mac.


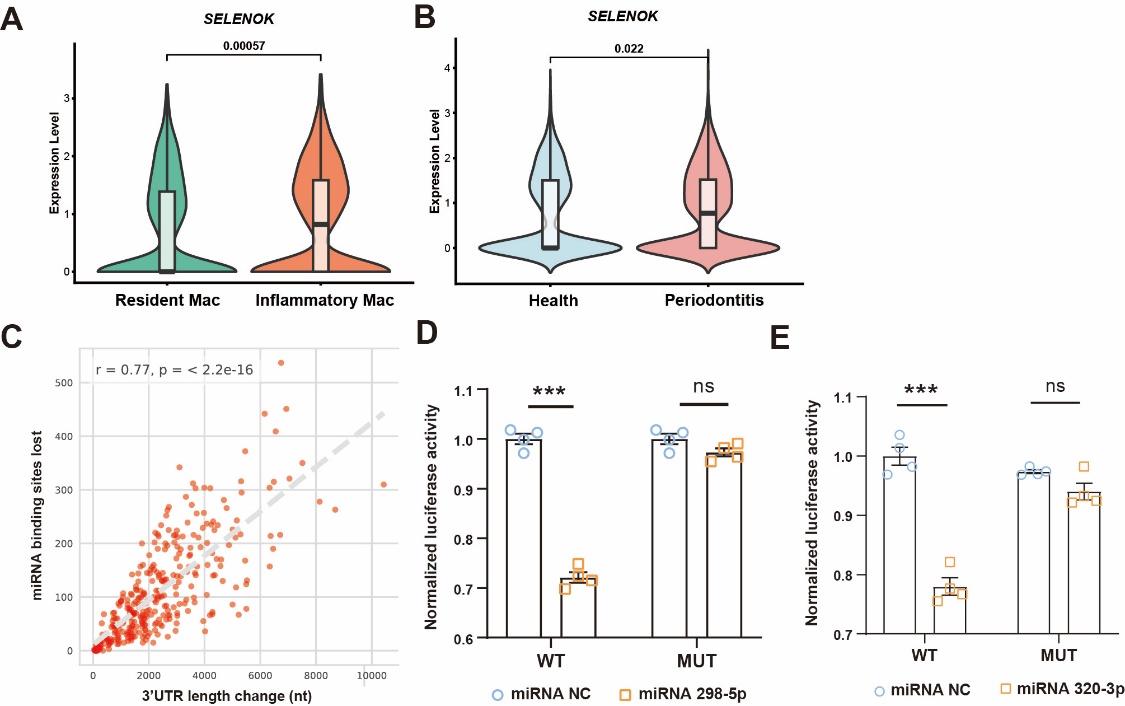


**Figure S5.**

(A) Violin plot of *SELENOK* expression in Inflammatory Mac versus Resident Mac. (B) Violin plot of *SELENOK* expression in macrophages: healthy versus periodontitis. (C) Scatter plot displaying 3’UTR length change and miRNA binding sites lost. (D) Bar chart of dual luciferase reporter assay for miRNA 298-5p. (E) Bar chart of dual luciferase reporter assay for miRNA 320-3p.

Table S1. Up-regulation miRNAs identified by small RNA sequencing in Periodontitis vs Control.

| **sRNA** | **Control** | | **Periodontitis** | **log2FoldChange** | **P value** |
| --- | --- | --- | --- | --- | --- |
| mmu-miR-135b-3p | 1.39E-17 | | 22.07753 | 7.492452055 | 8.28E-06 |
| mmu-miR-540-3p | 1.39E-17 | | 17.82934 | 7.186236035 | 7.55E-05 |
| mmu-miR-501-5p | 1.39E-17 | | 14.43078 | 6.883710415 | 0.000315 |
| mmu-miR-350-5p | 1.39E-17 | | 11.88185 | 6.606218092 | 0.001437 |
| mmu-miR-652-5p | 1.39E-17 | | 10.18255 | 6.386291609 | 0.002435 |
| mmu-miR-5113 | 1.39E-17 | | 7.6336 | 5.976991628 | 0.012731 |
| mmu-miR-351-3p | 1.39E-17 | | 6.783943 | 5.809926852 | 0.022677 |
| mmu-miR-489-3p | 1.39E-17 | | 6.783943 | 5.809926852 | 0.022677 |
| mmu-miR-200c-5p | 1.39E-17 | | 5.934282 | 5.620950894 | 0.040965 |
| mmu-miR-450a-1-3p | 1.39E-17 | | 5.934282 | 5.620950894 | 0.040965 |
| mmu-miR-6933-5p | 1.39E-17 | | 5.934282 | 5.620950894 | 0.040965 |
| mmu-miR-21a-3p | 2.368715 | | 29.72426 | 3.591381956 | 0.000112 |
| mmu-let-7j | 3.545605 | | 43.3184 | 3.571838112 | 6.09E-06 |
| mmu-miR-296-3p | 2.368715 | | 25.47608 | 3.36998253 | 0.000419 |
| mmu-miR-25-5p | 2.368715 | | 19.52862 | 2.988767124 | 0.003385 |
| mmu-miR-452-5p | 1.19185 | | 10.18255 | 2.988332023 | 0.021567 |
| mmu-miR-877-5p | 3.545605 | | 28.02499 | 2.94603908 | 0.000466 |
| mmu-miR-679-5p | 2.368715 | | 17.82934 | 2.858384247 | 0.006966 |
| mmu-miR-5620-5p | 1.19185 | | 8.483254 | 2.728742866 | 0.034867 |
| mmu-miR-342-5p | 3.545605 | | 22.92717 | 2.657936146 | 0.003149 |
| mmu-miR-6539 | 3.545605 | | 22.92717 | 2.657936146 | 0.003149 |
| mmu-miR-494-3p | 45.91695 | | 266.7715 | 2.5358296 | 1.88E-07 |
| mmu-miR-188-5p | 3.545605 | | 18.67898 | 2.364232949 | 0.011803 |
| mmu-miR-667-5p | | 3.545605 | 18.67898 | 2.364232949 | 0.011803 |
| mmu-miR-326-3p | | 9.430409 | 47.56657 | 2.322128374 | 0.000416 |
| mmu-miR-17-3p | | 20.02327 | 88.34892 | 2.135938357 | 0.000125 |
| mmu-miR-145a-5p | | 3578.039 | 14763.17 | 2.044731152 | 1.66E-06 |
| mmu-miR-1193-3p | | 7.076457 | 28.87462 | 2.013480332 | 0.009731 |
| mmu-miR-147-5p | | 12.96136 | 50.11547 | 1.942892312 | 0.002011 |
| mmu-miR-185-3p | | 4.722537 | 17.82934 | 1.894758552 | 0.033376 |
| mmu-miR-582-3p | | 67.10266 | 225.1395 | 1.744914481 | 0.000198 |
| mmu-miR-410-3p | | 9.430409 | 31.42353 | 1.726141358 | 0.013705 |
| mmu-miR-182-3p | | 16.49231 | 54.36363 | 1.714970007 | 0.003629 |
| mmu-miR-222-3p | | 640.2929 | 2079.884 | 1.699548693 | 6.73E-05 |
| mmu-miR-92a-3p | | 3377.952 | 10855.72 | 1.684206259 | 6.59E-05 |
| mmu-miR-223-3p | | 289.5523 | 918.4383 | 1.665031581 | 0.000122 |
| mmu-miR-142a-3p | | 416.6663 | 1288.877 | 1.628926872 | 0.000146 |
| mmu-miR-142b | | 416.6663 | 1288.877 | 1.628926872 | 0.000146 |
| mmu-miR-423-3p | | 729.7435 | 2214.125 | 1.601149533 | 0.000165 |
| mmu-miR-744-3p | | 8.25343 | 24.62644 | 1.566206083 | 0.042814 |
| mmu-miR-301a-5p | | 14.13834 | 41.61914 | 1.551285958 | 0.015487 |
| mmu-miR-1983 | | 80.04947 | 233.6358 | 1.544180585 | 0.000855 |
| mmu-miR-339-5p | | 74.16456 | 214.0943 | 1.528248902 | 0.00101 |
| mmu-miR-298-5p | | 11.78437 | 33.97243 | 1.52000114 | 0.021872 |
| mmu-miR-212-3p | | 7.076457 | 20.37826 | 1.5135338 | 0.044489 |
| mmu-miR-10a-5p | | 1321.765 | 3698.43 | 1.484381628 | 0.000425 |
| mmu-miR-99a-3p | | 10.60739 | 29.72426 | 1.478442648 | 0.033174 |
| mmu-miR-351-5p | | 207.1636 | 576.8867 | 1.477101782 | 0.000696 |
| mmu-miR-487b-3p | | 9.430409 | 26.32572 | 1.471975332 | 0.041904 |
| mmu-miR-369-3p | | 24.73122 | 67.10812 | 1.436745956 | 0.007809 |
| mmu-miR-135b-5p | | 220.1104 | 593.8793 | 1.431561505 | 0.000984 |
| mmu-miR-329-5p | | 80.04947 | 214.0943 | 1.418242182 | 0.002175 |
| mmu-miR-30c-2-3p | | 303.6761 | 772.3018 | 1.346369745 | 0.001757 |
| mmu-miR-199b-5p | | 2229.218 | 5643.235 | 1.339949152 | 0.001376 |
| mmu-miR-362-5p | | 164.7922 | 413.7576 | 1.327661401 | 0.002564 |
| mmu-miR-193b-3p | | 41.20901 | 101.0934 | 1.292796182 | 0.01019 |
| mmu-miR-21a-5p | | 388251 | 937421.5 | 1.271708047 | 0.002265 |
| mmu-miR-345-3p | | 92.99628 | 216.6432 | 1.219297193 | 0.007949 |
| mmu-miR-146b-5p | | 3614.526 | 8367.152 | 1.210910339 | 0.003684 |
| mmu-miR-370-3p | | 69.45662 | 160.5676 | 1.207962722 | 0.010173 |
| mmu-miR-125b-1-3p | | 343.6935 | 785.0463 | 1.191449474 | 0.005208 |
| mmu-miR-221-5p | | 410.7814 | 920.1376 | 1.163310562 | 0.006286 |
| mmu-miR-147-3p | | 22.37725 | 50.11547 | 1.160141069 | 0.042612 |
| mmu-miR-676-3p | | 103.5891 | 226.8388 | 1.130147425 | 0.012631 |
| mmu-miR-205-5p | | 51548.29 | 111031.4 | 1.106970689 | 0.007656 |
| mmu-miR-503-5p | | 76.51852 | 163.1165 | 1.091176901 | 0.018793 |
| mmu-miR-708-5p | | 861.5655 | 1784.212 | 1.05018302 | 0.012232 |
| mmu-miR-28a-3p | | 417.8433 | 864.9116 | 1.049442561 | 0.013504 |
| mmu-miR-532-3p | | 41.20901 | 84.10076 | 1.027690162 | 0.044151 |
| mmu-miR-132-3p | | 77.69551 | 158.0187 | 1.023417261 | 0.02694 |
| mmu-miR-155-5p | | 441.383 | 896.3479 | 1.021891922 | 0.015888 |
| mmu-miR-16-1-3p | | 221.2873 | 439.2465 | 0.988848702 | 0.022442 |
| mmu-miR-320-3p | | 1545.392 | 2945.658 | 0.930582235 | 0.025406 |
| mmu-miR-125a-5p | | 2452.845 | 4665.31 | 0.927495282 | 0.025528 |
| mmu-miR-34c-5p | | 915.7066 | 1730.685 | 0.918328462 | 0.028082 |
| mmu-miR-541-5p | | 1560.693 | 2944.808 | 0.91595334 | 0.027761 |
| mmu-miR-221-3p | | 2568.189 | 4836.086 | 0.91306812 | 0.027843 |
| mmu-miR-93-5p | | 3143.733 | 5835.252 | 0.892300008 | 0.031418 |
| mmu-miR-210-3p | | 460.2147 | 852.1671 | 0.888719305 | 0.035646 |
| mmu-miR-96-5p | | 2789.462 | 5152.149 | 0.885169493 | 0.032853 |
| mmu-miR-31-5p | | 3159.034 | 5602.453 | 0.826560702 | 0.046023 |
| mmu-miR-532-5p | | 1949.097 | 3441.842 | 0.820351869 | 0.048137 |
